# Obesity-compromised immunity in post-COVID-19 condition: a critical control point of chronicity

**DOI:** 10.3389/fimmu.2024.1433531

**Published:** 2024-08-12

**Authors:** Soonwoo Jang, Wooyoung Hong, Yuseok Moon

**Affiliations:** ^1^ Laboratory of Mucosal Exposome and Biomodulation, Department of Integrative Biomedical Sciences, Pusan National University, Yangsan, Republic of Korea; ^2^ Department of Medicine, Pusan National University, Yangsan, Republic of Korea; ^3^ Biomedical Research Institute, Pusan National University Hospital, Yangsan, Republic of Korea; ^4^ Department of Chemistry and Chemical Biology, Harvard University, Cambridge, MA, United States; ^5^ Graduate Program of Genomic Data Sciences, Pusan National University, Yangsan, Republic of Korea

**Keywords:** COVID-19, SARS-CoV-2, long COVID, post COVID-19 condition, obesity, immunity, ACE2, adipose tissue

## Abstract

Post-COVID-19 condition is recognized as a multifactorial disorder, with persistent presence of viral antigens, discordant immunity, delayed viral clearance, and chronic inflammation. Obesity has emerged as an independent risk factor for both SARS-CoV-2 infection and its subsequent sequelae. In this study, we aimed to predict the molecular mechanisms linking obesity and post-COVID-19 distress. Viral antigen-exposed adipose tissues display remarkable levels of viral receptors, facilitating viral entry, deposition, and chronic release of inflammatory mediators and cells in patients. Subsequently, obesity-associated inflammatory insults are predicted to disturb cellular and humoral immunity by triggering abnormal cell differentiation and lymphocyte exhaustion. In particular, the decline in SARS-CoV-2 antibody titers and T-cell exhaustion due to chronic inflammation may account for delayed virus clearance and persistent activation of inflammatory responses. Taken together, obesity-associated defective immunity is a critical control point of intervention against post-COVID-19 progression, particularly in subjects with chronic metabolic distress.

## 1 Introduction: obesity and SARS-CoV-2 infection

Established risk factors for SARS-CoV-2 infection include advanced age, diabetes, and metabolic syndrome (1). Obesity has been associated with an increased risk of SARS-CoV-2 infection, as well as an increased likelihood of needing intensive care and invasive mechanical ventilation. Obesity emerged as the predominant comorbidity among healthcare workers who were hospitalized in 14 US states, as reported by the COVID-19-associated Hospitalization Surveillance Network (2). Obesity has been recognized as an independent risk factor for the onset of COVID-19 (3). Although there appears to be a non-linear correlation between body mass index (BMI) and the severity of COVID-19 (4), multiple studies have indicated that individuals with a BMI over 25 kg/m2 experienced more severe illness and higher mortality rates compared to those with a BMI under 25 kg/m2. Excessive adiposity has been identified as a risk factor for the severity and mortality associated with SARS-CoV-2 infection (5). Abdominal adiposity, rather than body mass index (BMI), is a more accurate indicator of the necessity for intensive care unit (ICU) admission and invasive mechanical ventilation in patients with COVID-19 (6). The ratio of visceral adipose tissue (VAT) to subcutaneous adipose tissue (SAT) is another promising predictor of adverse outcomes in hospitalized patients with COVID-19 (7, 8).

In addition to its impact on the acute phase of SARS-CoV-2 infection, several studies have examined the association between post-COVID condition and obesity. In a cohort of 486,149 non-hospitalized patients, obesity was identified as a risk factor for both SARS-CoV-2 infection and post-COVID condition (9). Obese patients had higher rates of chronic pain, poorer sleep quality, and more non-specific post-COVID-19 symptoms after hospital discharge compared to non-obese individuals (10, 11). Furthermore, a significant association between obesity and post-COVID-19 condition was observed in hospitalized patients (12). Obesity, older age, female sex, smoking, COVID-19 hospitalization, socioeconomic deprivation, and healthcare employment were all correlated with higher odds of persistent symptoms in a cohort of 508,707 individuals in England (13).

In this review, we aimed to explore the molecular mechanisms underlying the association between obesity and post-COVID-19 condition. While numerous studies have investigated the relationship between obesity and the acute phase of COVID-19, the mechanisms linking obesity to the development of post-COVID-19 condition remain unclear. To address this gap, we synthesized findings from various studies, encompassing both *in vivo* and *in vitro* research. Our hypothesis posits that understanding these molecular mechanisms could elucidate the pathways through which obesity predisposes individuals to the development of post-COVID-19 condition. By examining a diverse array of studies, we aimed to provide insights into the intricate interplay between obesity and the long-term consequences of COVID-19 infection.

## 2 Viral entry into adipose tissues

### 2.1 Tissue tropism: adipose tissues as the SARS-CoV-2 reservoir

Adipose tissue has been implicated as a reservoir for viral spread and cytokine amplification of SARS-CoV-2 (14). Evidence of viral replication within the adipose tissue of *in vivo* experimental animals has also been reported for viruses such as HIV, SIV, and H1N1 influenza A (15–17). SARS-CoV-2 is widely distributed, predominantly among patients who died from severe COVID-19, and virus replication is present in multiple respiratory and non-respiratory tissues, including the brain, early in infection (18–21). Several autopsy studies have investigated SARS-CoV-2 infection in the lungs and extrapulmonary organs. Gastric and gallbladder tissues can even retain SARS-CoV-2 particles for more than one year after the resolution of COVID-19 (22). Postmortem studies identified SARS-CoV-2 localized within the adipose tissue, although in which cells were not always clear. The studies on patients with severe COVID-19 indicate a notable presence of SARS-CoV-2 RNA in adipose tissues (21, 23–25). Specifically, SARS-CoV-2 RNA was detected in 48 out of 110 subcutaneous adipose tissue (SAT) samples, as reported across four different patient cohorts (21, 23–25). In particular, two of these studies found detectable levels of SARS-CoV-2 RNA in 13 of 33 visceral adipose tissue (VAT) samples (23, 25). All of the evidence suggests that SARS-CoV-2 may indeed have the capability to infect adipose tissue. The average turnover rate of adipocytes is estimated to be about 8% per year (26). In this respect, adipose tissue has been implicated as a good environment for replication and persistence. SARs-CoV-2 viral RNA persistence in tissues has been associated with the severity and long-term sequelae of several other RNA virus infections (27). Another clinical assessment demonstrated that higher levels of plasma-free fatty acids were observed in individuals with COVID-19 compared to a control group, indicating a close association between elevated basal lipolysis of triglycerides in adipose tissue and viral infection (28). Furthermore, elevated levels of fatty acids and triglyceride accumulation boosted the replication of SARS-CoV-2 in human cells (29). Therefore, inhibition of lipolysis using atorvastatin and tetrahydrolipstatin remarkably retarded viral replication and attenuated viral receptor expression (23). All of the evidence suggests that the lipid-laden nature of adipocytes and elevated fatty acid levels due to increased lipolysis play pivotal roles in enhancing SARS-CoV-2 replication.

However, it is still unclear which cells in the adipose tissue are susceptible to infection. Adipose tissue cells can be divided into adipocytes and stromal vascular fractions (SVF). SVF comprises endothelial cells, macrophages, lymphoid cells, and fibroblasts (30). The precise mechanism of entering SARS-CoV-2 into adipose tissue has been controversial. *In vitro* cell studies have found that SARS-CoV-2 can infect mature, differentiated adipocytes and macrophages *in vitro* but not preadipocytes (24, 25). Another study showed that increased infiltration of macrophages due to obesity could be the pathway to infect adipose tissue by the SARS-CoV-2 virus in a clinical setting (31). In a single-cell level study using adipose tissue samples from patients undergoing bariatric and cardiothoracic surgery, SARS-CoV-2 RNA was detected in various adipose depots and within the cytoplasm of adipocytes. Furthermore, SARS-CoV-2 can directly infect both human and mouse adipocytes, exhibiting efficient multi-cycle replication in mature differentiated adipocytes rather than immature adipocytes. This tissue tropism and deposition are notably specific in the infection process of SARS-CoV-2, compared with the Influenza A virus (H1N1) (23).

Human adipose tissues from COVID-19 cases displayed leukocyte infiltration and upregulation of immune cell markers such as CD45 (pan-leukocyte), CD3 (T-cells), CD57 (natural killer cells), and CD68 (macrophages) in addition to nucleocapsid antigen of SARS-CoV-2 (21, 25). The presence of the virus, combined with protective adipokines, contributes to both local and systemic inflammation, thereby exacerbating the post-infection progression in subjects with obesity (25, 32). Adipose tissue-derived adipokines and chemokines activate macrophages and their release of proinflammatory cytokines through Toll-like Receptor 4-linked transcriptional modulation (33, 34). In particular, monocyte chemotactic protein-1 triggers recruitment and accumulation of virus-exposed macrophages in the adipose tissues (35). Taken together, there could be two main pathways of viral entry into the adipose tissues. Mature adipocytes are permissive to SARS-CoV-2 infection and are involved in production of inflammatory mediators and leukocyte recruitment. In addition to the adipocyte infection, the infiltration of abortively infected macrophages results in increased viral load and chronic inflammatory distress in adipose tissues.

### 2.2 Obesity-associated susceptibility to receptor-mediated viral entry

SARS-CoV-2 is a single-strand, positive-sense RNA genome virus, belonging to the Coronaviridae family; it is characterized by an envelope, on the surface of which are located projections known as spikes (36). In particular, spike protein consists of S1 and S2 subunits: the interaction between the S1 subunit and angiotensin-converting enzyme-2 (ACE2) receptors grants entry of the virus into the host cells. This process is crucial, as S-protein-RNA-based vaccines have been developed against SARS-CoV-2. SARS-CoV-2 enters the target cell, binding to the receptor protein ACE2, which is located in many human tissues, such as the lungs, heart, and adipose tissue (37). Furthermore, ACE2 expression is enhanced by several proinflammatory cytokines the levels of which are already elevated in obese patients (38). In turn, the elevated expression of ACE2 in the adipose tissue of obese patients may determine greater viral entry and replication (14, 39), suggesting a role for adipose tissue as a virus reservoir to enhance viral spread.

Although the exact mechanisms of SARS-CoV-2 infection through the ACE2 were unclear, there was some experimental evidence that the ACE2 and its upstream, intragenic elements were closely associated with the entry of the virus. A lack of ACE2 in mice leads to a dramatic reduction in the replication of the SARS-CoV virus and much less severe pathological changes in the lungs compared to wild-type mice (40). ACE2 has been also reported to bind the 2019-nCoV S ectodomain with an affinity of approximately 15 nM, which is 10 to 20 times higher than the binding of ACE2 to SARs-CoV S (41). In contrast to transgenic mice in which human ACE 2 is expressed in cilia under a human FOXJ 1 promoter, mice in which ACE 2 is expressed in club cells under an endogenous ACE 2 promoter show a robust immune response to SARS infection, resulting in rapid viral clearance (42). Therapeutic administration of engineered ACE2 protected hamsters from SARS-CoV-2 infection and decreased lung virus titers and pathology (43). SARS-CoV-2 clearance was accelerated by the nasal spray of IgM-like ACE2 fusion protein in humans (44).

Obesity is characterized by hypertrophic expansion of adipose tissue due to augmented storage of lipids. Adipose tissue express ACE2 which is known as SARS-CoV-2 entry. ACE2 levels are notably elevated in the adipose tissue of obese individuals compared to those in lean individuals (45). White Adipose tissues expresses similar ACE2 levels to the lungs of the same individual (37). A recent study demonstrated that ACE2 gene expression is higher in visceral and subcutaneous adipose tissues than in lung, the major target organ of SARS-CoV-2 (46). During the COVID-19 pandemic, patients who required intensive care unit (ICU) admission or invasive mechanical ventilation (IMV) had greater VAT depots than those who did not (6), indicating a susceptibility of VAT to the viral entry. In particular, the expression of ACE2 is higher in VAT, rather than SAT (47). However, ACE2 mRNA is identified in differentiated preadipocytes and sporadically detected in mature adipocytes (23, 25). Since mature and differentiated adipocytes are permissive to SARS-CoV-2 infection, we cannot exclude the possibility of ACE2-independent viral entry into these adipocytes.

**Figure 1:**
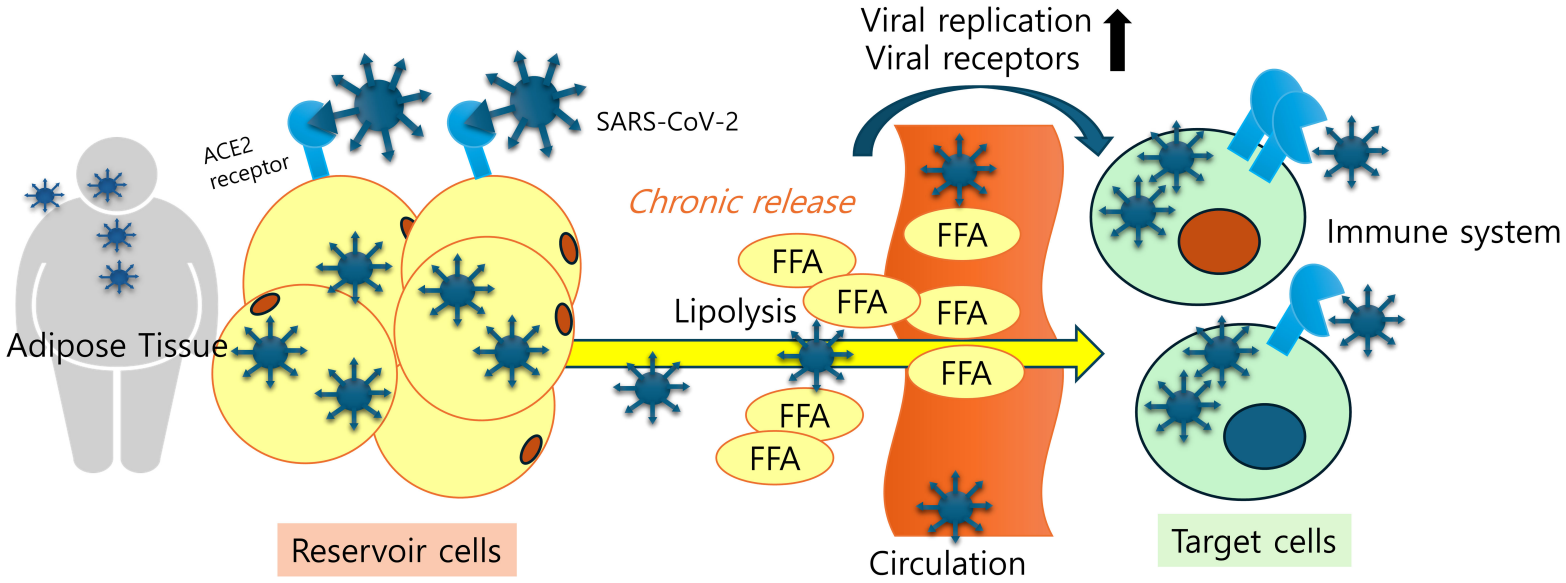
Adipose tissue tropism and a SARS-CoV-2 reservoir. Viral antigen-exposed adipose tissues display remarkable levels of viral receptors, which facilitates viral entry, deposition, and chronic release of inflammatory mediators and cells in patients. Lipid-laden nature of adipocytes and elevated fatty acid levels due to increased lipolysis play pivotal roles in enhancing SARS-CoV-2 replication and ACE2 expression.

The tropism of SARS-CoV-2, including pulmonary ACE2 expression and ACE2-expressing cell types, exhibited distinct sub-phenotypes associated with vulnerable demographics such as the sick, male, and older populations. Through the integration of multiple COVID-19 cohorts in genome-wide association studies (GWASs) and cis-expression quantitative trait loci (cis-eQTLs) of ACE2, Mendelian randomization (MR) analyses demonstrated that ACE2 played a causal role in both COVID-19 susceptibility and severity (48). In the immunohistochemistry results of post-mortem lung tissues, patients with severe SARS-CoV-2 showed a significant up-regulation of the expression level of ACE2, CD163, and CD61 compared to the control group. ACE2 expression demonstrated a significant association with the levels of coagulation and inflammation markers (CD163 and CD61) in linear regression analysis. These post-mortem lung tissues displayed diffuse alveolar damage, acute bronchopneumonia, and acute lung injury with SARS-CoV-2 viral protein detected in a subset of cases (49). Despite the controversy surrounding the mechanisms of SARS-CoV-2 entry into adipose tissue via the ACE2 receptor, human ACE2 expression and its upstream elements may contribute to the viral reservoir and chronic outcomes of the viral antigens leading to systemic distress (**Figure 1**).

## 3 Discordant immunity and viral clearance in chronic inflammation and metabolic distress

Post-COVID-19 condition has been defined by the WHO Delphi consensus and the UK National Institute for Health and Care Excellence (NICE) as occurring in individuals with a history of probable or confirmed SARS-CoV-2 infection, usually 3 months after COVID-19 onset, with symptoms lasting at least 2 months that cannot be explained by an alternative diagnosis. Common symptoms include fatigue, shortness of breath, cognitive dysfunction, and other symptoms that generally have an impact on the functioning of daily life (50). Persistent inflammation is hypothesized to play a key role in the pathophysiology of long COVID (51).

### 3.1 Inflammatory mediators in virus-exposed adipose tissues

Obesity is a chronic inflammatory condition characterized by systemic metabolic dysregulation, including insulin resistance, elevated lipid concentrations, abnormal adipose tissue distribution, and altered adipokine profiles, particularly with elevated leptin and reduced adiponectin levels, along with a persistent state of low-grade inflammation (52, 53). It is widely recognized that obesity, distinguished by atypical levels of body fat, is linked to numerous complications, such as hypertension, dyslipidemia, type 2 diabetes (T2D), and immune dysfunction (54, 55). In obesity, adipose tissue experiences alterations characterized by an augmentation in both the size and quantity of adipocytes, accompanied by the infiltration of immune cells. Immunosuppression and disproportionate cytokine activation are exacerbated by the chronic low-grade inflammatory characteristic of obesity, which is characterized by increased activation of circulating cytokines, particularly Interlukin-6 (IL-6). In humans, elevated circulating inflammatory mediators such as IL-6 and markers such as high-sensitivity C-reactive protein correlate positively with obesity (56). IL-6, similar to Tumor Necrosis Factor-alpha (TNF-α), is secreted by both adipocytes and macrophages. It inhibits the activity of lipoprotein lipase, thereby contributing to the dysfunction of fat storage in adipose tissue (57). The generation of oxidants and glycation products can indirectly impair immune function and trigger inflammation by promoting the release of effector cytokines, including TNF-α and interferon-gamma (IFN-γ). These cytokines subsequently contribute to the dysregulation of pro-inflammatory immune cells, such as Type 1 T helper (Th1) cells, macrophages, and dendritic cells. In addition to immune dysregulation, hypertrophic adipocytes play a significant role in fostering an inflammatory milieu by releasing cytokines such as IL-6 and adipokines.

Dysregulation of the immune response due to adipokines produced by adipose tissue could be hypothesized to account for the altered humoral response in obese individuals. Adipose tissue produces a large number of adipokines, which are signal molecules with a wide range of effects on many organ systems (58). Leptin is mainly secreted by white adipose tissue. Its levels are positively correlated with the amount of body fat. Leptin exerts a variety of other effects, such as regulation of immune function and inflammation, in addition to its role in regulating energy homeostasis and metabolism (59). High leptin levels are also linked to alveolar fluid accumulation and increased inflammation during hypoxia and ARDS (60). Leptin appears to have specific effects on helper T lymphocyte function by regulating the proliferation of cells involved in both innate and acquired immune responses (61, 62). *In vitro* assays have shown that the expression of some integrins (VLA-2, CD49b) and their ligands (ICAM-1, CD54) in CD4+ cells is induced by leptin, although no effects were found for other adhesion molecules such as CD49a,c,d, CD50, and CD62L (62).

Uncontrolled pro-inflammatory activation and severe condition in patients with COVID-19 has been connected to molecular changes triggered by hyperleptinemia which is the most common feature of obesity (63, 64). Leptin demonstrated strong consistency with CXCL-10 and TNF-α in predicting disease severity. Mechanistically, leptin synergistically increased the expression levels of inflammatory cytokines and surface markers in monocytes along with IL-6 (64). It also showed correlations with reduced lymphocyte counts and disease progression. Plasma levels of IL-2, TNF-α, and IL-4 are elevated during the acute phase of COVID-19. In post-COVID-19 condition, cytokine expression patterns change. Similar to the acute phase, higher levels of IL-2 and IL-17 are observed in individuals with long COVID-19. However, IL-4, known for its anti-inflammatory properties, is reduced in patients who develop post-COVID-19 sequelae compared to those who fully recover. Therefore, high serum levels of IL-2 and IL-17, along with low levels of IL-4 and IL-10, are closely associated with the chronic inflammatory distress in long COVID-19 (65, 66). While IL-2 promotes the proliferation and differentiation of CD8+ T cells, IL-4 is involved in adaptive immunity and plays a crucial role in the regulation of the immune system, which is controlled by activated T helper (Th) cells. The presence of IL-4 during the response of naive Th cells causes the precursors to develop into a population composed largely of “Th2-like” effectors secreting IL-4 and IL-5, but little IL-2 or IFN-γ (67). Moreover, obese individuals show notably reduced levels of adipose tissue eosinophils and eosinophil-derived IL-4 secretion, counteracting hyperleptinemia and insulin resistance (68). Therefore, viral infection-induced discordant immunity may contribute to the aggravation of metabolic homeostasis in the obese population.

### 3.2 Delayed viral clearance due to insufficient neutralizing antibodies

It has been demonstrated that the humoral immune responses play critical roles as determinants of viral clearance and protection during the acute phase of SARS-CoV-2 infection (69). Delayed clearance of the SARS-CoV-2 virus, including persistent viral infection or the presence of viral components, is also known to be closely associated with the symptoms of long COVID. There is some evidence to suggest that, at least in a subset of patients with long COVID, the symptoms may be associated with the persistence of the virus. Even at 1 year post-infection, the SARS-CoV-2 protein spike (S) was detected in 60% of long COVID patients but not controls; the more organ systems involved in symptoms, the greater the amount of detectable S. taken at face value, this is further evidence that long COVID symptoms may be due to some form of uncleared viral reservoir (70). Studies of post-mortem tissues provide a much broader list of potential sites for persistent viral reservoirs, including visceral adipose tissue (18, 20). Although most of the tissues studied were obtained from people who had acute, severe, and fatal COVID-19, it can be predicted that the chronic release of viral antigens from the adipose tissues could be important in adverse outcomes of the long COVID.

Obesity (but not diabetes) was associated with a 2-fold risk of developing influenza or viral infection-related illness. Despite more clinical reports of illness, no differences in antibody seroconversion rates or seroprotective titers were observed after vaccination in the normal-weight and obese cohorts (71). The differences in these studies may also reflect the timing of sampling. Some studies show a normal initial antibody titer response, but then a decreased antibody response in people with obesity when studied 12 months after vaccination (72). In a small study of 68 individuals with NAFLD, impaired immune responses to hepatitis B vaccines were reported in obese individuals with a BMI >35, with antibody titers measured after the third dose of the vaccine. The number of individuals demonstrating HBsAg-stimulated proliferation of CD4+ T cells was also lower in individuals with a BMI >35, as was the relative rate of T-cell proliferation (73, 74). The obese population was significantly associated with lower antibody titers after COVID-19 vaccination. This was due to a decreased antibody response to SARS-CoV-2 vaccines (75). Furthermore, 6 months after the second vaccine dose, 55% of individuals with severe obesity had unquantifiable titers of neutralizing antibodies against authentic SARS-CoV-2 virus compared to 12% of individuals with normal BMI (P=0.0003). After a third vaccine dose, individuals with severe obesity showed a more rapid decline in neutralizing capacity (76). Another study measuring human IgG levels specific to SARS-CoV-2 spike and nucleocapsid (NP) proteins, similarly revealed a significant reduction in both the rate and titer of anti-SARS-CoV-2 neutralizing antibodies among vaccinated obese individuals in comparison to the controls (77).

Studies of natural human infection have shown that SARS-CoV-2 infected individuals can produce potent neutralizing Abs targeting the SARS-CoV-2 S protein. *In vitro* and *in vivo*, these neutralizing Abs inhibit infection by SARS-CoV-2 (78). This humoral immunity was the key to successful protection against the virus and vaccines such as BNT162b2 and mRNA-1273 have been produced targeting these neutralizing Abs (79). Immunoglobulin alterations during the acute phase of SARS-CoV-2 infection have been associated with post-COVID-19 condition. Specifically, decreased titers of IgM and IgG3, along with elevated levels of autoantibodies, are linked to the development of Long-COVID symptoms (80). The levels of S-IgG were lower in patients with post-COVID-19 condition during follow-up, with differences reaching statistical significance at months 2 and 6 after discharge compared to non-symptomatic patients. At 12 months, the frequency of positive neutralizing antibodies was significantly lower in patients with post-COVID-19 condition, and titers of SARS-CoV-2 S1/S2 IgG tended to be lower (81). That is, the post-COVID-19 condition was closely related to the dysregulated immune response in hospitalized patients with decreased frequency of detectable neutralizing antibodies and decreased anti-spike antibody levels. Long-term COVID symptoms of any severity were reported by 9.5% of double-vaccinated study participants compared to 14.6% of socio-demographically matched unvaccinated participants. This represents a 41% reduction in the likelihood of long-term COVID at 12 weeks. Several studies have also investigated the possibility that vaccination may improve symptoms in people with a history of long COVID (82–84). The low titer of neutralizing antibodies worsens viral clearance. Delayed discharge from the hospital and delayed remission of symptoms were strongly associated with delayed viral clearance. Prolonged shedding of SARS-CoV-2 RNA was found to be independently linked with delayed hospital admission in a study involving 652 hospitalized patients (85, 86). Post-COVID-19 condition may be due to persistent SARS-CoV-2 reservoirs resulting from low viral clearance.

Obesity-associated low titers of neutralizing antibodies can be explained in terms of adipokine-mediated immune regulation. Leptin is a primarily proinflammatory adipokine that influences both innate and adaptive immune responses by stimulating the production of proinflammatory cytokines (IL-2, IFN-γ, TNF-α) and suppressing the production of humoral immunity-facilitating cytokines (IL-4 and IL-5). Blocking experiments showed that IL-4, although undetectable in culture supernatants, was critically involved in the induction of B cell proliferation and Ig secretion *in vivo* and *in vitro* (87). Several studies suggest that only IL-4-producing clones (th2) support Ig synthesis by examining the helper activity of T cell lines (88, 89). IL-4 preferentially activates, proliferates, and differentiates B lymphocytes and promotes immunoglobulin E isotype. It therefore plays a crucial role in the induction of humoral immunity-regulating Th2 cells (90, 91). Moreover, IL-4 and IL-5 have been known to be associated with IgG1, IgE, and IgA synthesis (92–94). As a result of these mechanisms, leptin appears to have a specific effect on T-lymphocyte responses by differentially regulating the proliferation of naive and memory T cells (62). Specifically, leptin increased Th1 and suppressed Th2 cytokine production (95), which could mitigate the humoral immune response.

### 3.3 The deterioration of cellular immunity in obese and post-COVID condition

Although cell-mediated immunity plays a critical role in the clearance of viruses, including SARS-CoV-2, obesity may deteriorate the quality of cellular immunity. Zucker rats are genetically obese animals because they lack the leptin receptor. Zucker rats showed lymphopenia (low levels of CD4+ and CD8+) in the thymus, spleen, and peripheral blood (96). Genetically obese strains have attenuated levels of macrophage phagocytic activity and proinflammatory cytokines (95). Macrophages from either ob/ob mice or db/db mice (animals lacking leptin or its receptor, respectively) are less active in the destruction of *Candida* than those isolated from lean control animals, suggesting a role for leptin in the phagocytic process (97). The impairment in the phagocytic activity of macrophages in genetically obese animals may be associated with the high levels of TNF-α, which are known to alter cytokines production (98). In humans, 38% of obese children and adolescents showed variable impairment of cell-mediated immune responses, such as delayed cutaneous hypersensitivity, abnormal lymphoproliferative responses to mitogens, and a reduction in the intracellular bacterial killing capacity of polymorphonuclear leukocytes (99). Obesity is associated with elevated leukocyte and lymphocyte subsets counts (except for NK and cytotoxic/suppressor T cells), lower T and B cell mitogen-induced lymphocyte proliferation, which were accompanied by higher monocyte and granulocyte phagocytosis as well as oxidative burst activity, but normal function of NK cells (100). CD8+ cytotoxic T cells and CD4+ T helper 1 (Th1) cells are the major components of cell-mediated antiviral defense. Th1 cell-derived cytokines, including IL-2 and IFN-γ, contribute to NK cell activation as well as the development of cytotoxic T lymphocyte (CTL) precursors into effectors. NK cells play a critical role in host defense until a specific cytotoxic T lymphocyte response is established (101).

**Figure 2:**
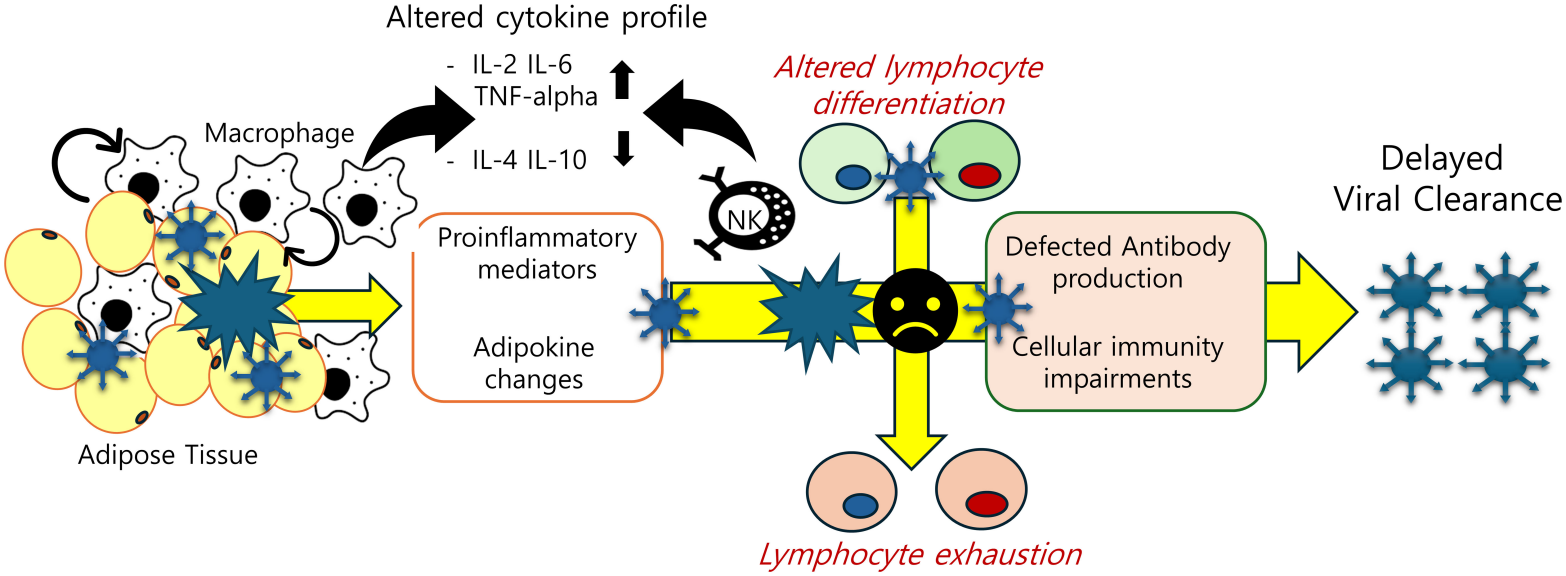
Adipose tissue-associated immune cell dysfunction in post-SARS-CoV-2 condition. Obesity-associated inflammatory insults are predicted to disturb cellular and humoral immunity by triggering abnormal cell differentiation and lymphocyte exhaustion. The decline in SARS-CoV-2 antibody titers and T-cell exhaustion due to chronic inflammation account for delayed clearance of the virus and persistent activation of inflammatory responses.

Adaptive immune cells (T cells) may play an important role in the propagation of adipose tissue inflammation in obesity. T cells from obese/T2D patients express lower levels of costimulatory molecules (CD69, CD28, CD40 ligand) and interleukin-12 receptor, and produce lower levels of interferon-γ and granzyme B, compared to healthy individuals (102, 103). Obesity-induced adipose tissue T cells have functional characteristics and gene expression resembling T cell exhaustion induced by local soluble factors and cell-to-cell interactions in adipose tissue in mice and human models (104). These immunological dysfunctions have been also observed in post-COVID condition patients. A triad of elevated IL-1β, IL-6, and TNF-α was proposed in a study investigating a diagnostic serum cytokine signature of chronic inflammation at 2 years post-infection (105). A cohort of patients with post-COVID condition exhibiting pulmonary sequelae characterized enriched populations of CD4+, CD8+ lymphocytes, and NK cells, along with upregulation of Granzyme (GZMB) and perforin at 12 months post-infection. High levels of CD8+ CD27- CD62L- cells, a short-lived effector subset, and low levels of CD8+ CD27+CD62L+ T naive central memory cells were observed. This subset of features were notable in continuous exposure to viral antigens with delayed clearance (106). Levels of cytotoxic CD8+ T cells were elevated and activated in patients with long-COVID 19 for up to 8 months after acute infection along with increased expression of TIM-3, PD-1, and soluble TIM-3 marker meaning exhaustion of T lymphocytes (107–109). In addition, another study using multiparameter flow to examine a long COVID cohort confirmed a profile consistent with T cell exhaustion (110). Multidimensional immune phenotyping focused on 99 non-hospitalized individuals with long COVID and a comparison cohort of 40 healthy recovering individuals. There was evidence of an activated B cell subset (CD86hi, HLA-DRhi), but increased PD1 and TIM3 expression on T cells and a decreased central memory CD4+ T cell subset (111, 112). Given the global up-regulation of PD-1 expression among T cells and the depletion observed within the naive subsets, there is a prevailing tendency to infer the reduction of immune cell populations as a result of viral infection. These observations have led to the hypothesis that T-cell exhaustion post-infection is a pivotal cause of long COVID and chronic inflammation in subjects with obesity. T-cell exhaustion could mechanistically account for the dysfunction of cellular immunity (**Figure 2**).

## 4 Discussion for translational perspectives

The post-COVID-19 condition is the persistence of symptoms following the infection for more than 3 months, which cannot be attributed to other specific diseases. The pathophysiology of the post-COVID-19 condition is complex and not fully understood, but it has been suggested that the prolonged immune response due to delayed clearance of the virus may play a role in its development (50). Delayed viral clearance has been consistently associated with delayed hospital discharge with persistent symptoms (86). In addition to the direct effects of SARS-CoV-2 long-term infection, effects on the autoimmune system and immunological changes resulting from delayed viral clearance have been implicated as potential reasons for the development of post-COVID-19 condition (50). These factors contribute to the complexity of the pathophysiology of the condition and underscore the need for further research to fully elucidate its mechanisms.

Obesity has been identified as an independent risk factor for the prognosis of SARS-CoV-2 infection (113). During the acute phase of SARS-CoV-2 infection, chronic inflammation associated with obesity has been implicated as a contributing factor to the cytokine storm observed in this infection. This pathophysiological mechanism is of particular concern due to its association with increased mortality and rates of admission to intensive care units (ICUs) during the acute phase of SARS-CoV-2 infection (114). Indeed, in addition to its impact on the acute phase of the disease, previous research has highlighted the association between obesity and the persistence of symptoms beyond the early phase of the illness. Studies investigating the long-term prognosis of the disease, including post-COVID-19 condition, have reported that obesity may contribute to the manifestation of persistent symptoms in individuals recovering from SARS-CoV-2 infection (51). The reasons why obesity may cause delayed viral clearance remain controversial, but several hypotheses have been proposed based on various studies. The first hypothesis is that obese individuals with hypertrophic adipose tissue have increased ACE2 expression, which facilitates the entry and deposition of SARS-CoV-2. Adipose tissue, which expresses ACE2 as much as the lung, appears to be anatomically and molecularly suitable as an entry point for SARS-CoV-2 into adipose tissue. As anatomical evidence, SARS-CoV-2 has been significantly detected by fluorescence immunohistochemistry in adipose tissue in post-mortem studies (115). In molecular studies, the human ACE2 has been demonstrated to exhibit significantly higher binding affinity to SARS-CoV-2 compared to mice (42–44).

High visceral fat is clinically proven to be associated with a poor prognosis following SARS-CoV-2 infection (47). VAT exhibits more ACE2 levels than SAT, resulting in a remarkably higher viral load in VAT (24). In addition to ACE2 levels, histopathologic findings from COVID-19 cases showed leukocyte infiltration and upregulation of the IFN-alpha pathway in adipose tissues. IFN-alpha levels are positively associated with transcription levels of the *ACE2* gene (21). In addition, cell surface markers such as CD45 (pan-leukocyte), CD3 (T-cells), CD57 (natural killer cells), and CD68 (macrophages) are detectable in the fat tissues. Moreover, VAT has been shown to secrete pro-inflammatory cytokines such as IL-6, TNF-α, and IL-1β (116–118). High expressions of inflammatory signatures including CC chemokine receptor 2, macrophage migration inhibitory factor, and monocyte/macrophage markers CD163 and CD14 makes VAT more susceptible to SARS-CoV-2 than SAT (119). Contrary to the findings mentioned above, there are also reports suggesting that viral entry into mature adipocytes by SARS-CoV-2 may occur independently of the ACE2-linked pathway (25). There has also been a report indicating a negative correlation between molecular levels of ACE2 expression and COVID-19 fatality in a population study (120). However, based on existing studies, adipose tissue is thought to play a role in viral entry and deposition, thereby contributing to delayed viral clearance. This delay in clearance is associated with persistent symptoms of SARS-CoV-2 infection (23). Second, the chronic inflammation induced by obesity may indeed lead to impairments in acquired immunity, affecting both humoral and cellular responses. These mechanisms could be implicated in delayed clearance of the virus. In a recent prospective cohort study, individuals with severe immunosuppression due to hematologic malignancy or transplant (S-HT) were found to have evidence of increased intra-host viral evolution and prolonged median times for nasal viral RNA and culture clearance. These findings correlated with resistance to antibody therapy. Additionally, these individuals exhibited impaired humoral and T-cell responses (121).

Obesity has also been associated with impaired immune responses to vaccines such as influenza, tetanus, and hepatitis B (72–74, 122). In addition, a significant decrease in neutralizing antibodies against authentic SARS-CoV-2 virus has been observed in obese individuals compared to normal-weight individuals, particularly evident six months after the second vaccine dose (76, 77). Similarly, impairments in the humoral immune response have been reported in post-COVID-19 patients, mirroring the findings observed in obese individuals with SARS-CoV-2 infection (123). In addition, the direct cellular immune response mediated by memory T cells may be impaired in obese individuals due to T cell exhaustion. Elevated levels of cytokines such as IL-6 and TNF-α, characteristic of the chronic inflammatory state associated with obesity, may contribute to the impairment of CD8+ T cells, leading to increased expression of the exhaustion markers PD-1 and TIM3 (104, 110, 111). This mechanism is closely related to the impaired cytotoxic T-cell immune response. Therefore, we believe that the impairments of acquired immunity could be closely associated with the persistent symptoms after COVID-19 infection. This is because low titers of antibodies and impaired T cell-mediated cytotoxic immune responses may not be sufficient to achieve clearance of the virus.

Extensive evaluations suggested evident associations between adiposity and delayed viral clearance. Furthermore, some interventions could ameliorate the pathophysiological status of obesity and post-COVID-19 condition. First, improving obesity through physical activity and dietary regulation is associated with enhanced immunologic recovery and mitigation of chronic inflammatory conditions (124, 125). For example, the adoption of ketogenic and Mediterranean diets, combined with regular physical activity, was proposed as a potential dietary therapy for obese individuals to prevent post-COVID-19 condition (126). Moreover, weight loss achieved through hypocaloric and very low-carbohydrate diets can improve adaptive immune responses elicited by SARS-CoV-2 mRNA vaccines in obese patients (127). Acute exercise was demonstrated to enhance macrophage phagocytic function in both lean and obese individuals compared with the sedentary controls (128). Additionally, the exercise promotes the production of interleukin-10 (IL-10), an anti-inflammatory cytokine, in adipocytes and stromal vascular cells (129). Secondly, several medicinal interventions associated with metabolic syndrome can be another option for preventing and resolving long COVID. For instance, outpatient treatment with metformin has demonstrated a reduced incidence of long COVID by approximately 41% in a randomized controlled study targeting adults aged 30-85 with COVID-19 and overweight or obesity (130). Additionally, using statins, a classical approach to suppressing lipolysis, or employing newly investigated inhibitors of VPS34 could be considered crucial in suppressing virus replication by reducing adipocyte lipolysis (23, 28, 131). Furthermore, from a symptomatic treatment perspective, medications like paracetamol and non-steroidal anti-inflammatory drugs can be utilized to manage specific symptoms such as fever and myalgia (132, 133). However, there were several limitations in the present prediction of obesity-mediated susceptibility to viral infection-induced diseases. First, most studies on the association between obesity and post-COVID-19 condition are based on animal experiments. There remains a scarcity of clinical studies that delve into the detailed investigation of immunologic molecules. Moreover, clinical studies with large cohorts are typically limited in their ability to measure all desired immunologic outcomes. By including different types of studies, we can maximize our understanding of the complex immunological landscape associated with the phenomenon under investigation. This inclusive approach allows for a comprehensive exploration of the topic, despite potential limitations in directly correlating findings between different study types. Second, in post-COVID-19 clinical studies, subjects are typically required to be followed for a longer period, usually at least three months. This extended follow-up duration can lead to loss of follow-up, which in turn makes it challenging to observe all immunologic markers in a clinical setting. Therefore, the molecular mechanisms underlying the association between obesity and post-COVID condition were not feasible to demonstrate in a large cohort clinical trial. Third, there are still numerous molecular pathways that remain to be elucidated. Both obesity and post-COVID-19 are known for their complexity in terms of understanding their mechanisms. In conclusion, we propose that the association between post-COVID-19 condition and obesity can be understood through the characteristics of adipose tissue, which may serve as a reservoir for the SARS-CoV-2 virus due to the expression of ACE2 receptors. In addition, the chronic inflammation induced by obesity likely plays a role in impairing immunity, thereby hindering clearance of the SARS-CoV-2 virus or its particles. This delayed viral clearance could potentially contribute to the development of post-COVID-19 condition.
